# A Task-Prompt-Guided Dual-Decoder Framework with Large Language Models for Multi-Phase Contrast CT Synthesis from Non-Contrast CT

**DOI:** 10.3390/jimaging12080365

**Published:** 2026-08-08

**Authors:** Liang Lyu, Hao Sun, Jiaqing Liu, Fang Wang, Lanfen Lin, Yen-Wei Chen

**Affiliations:** 1 Graduate School of Information Science and Engineering, Ritsumeikan University, Ibaraki 567-8570, Osaka, Japan; 2 Department of Radiology, SRRSH of School of Medicine, Zhejiang University, Hangzhou 310016, China; 3 College of Artificial Intelligence, Zhejiang University, Hangzhou 310058, China

**Keywords:** non-contrast CT, multi-phase contrast CT synthesis, large language model, task prompt, dual-decoder network, medical image synthesis

## Abstract

Synthesizing multi-phase contrast-enhanced CT images from non-contrast CT (NCCT) may provide complementary phase-specific cues for preliminary assessment. After rigorous clinical validation, such images could provide clinical decision support or aid diagnostic triage by identifying cases that warrant further acquired contrast-enhanced CT (CECT) work-up; they are not intended to replace acquired CECT. Arterial-phase (ART) and portal-venous-phase (PV) images share anatomical structures but exhibit distinct enhancement patterns and intensity distributions, which makes simultaneous multi-phase synthesis challenging for conventional single-decoder models. We propose a task-prompt-guided dual-decoder framework that combines a shared Swin Transformer encoder, two phase-specific decoders, learnable task prompts initialized from Qwen3-8B semantic representations, two phase-specific adversarial discriminators, and an independent ART/PV domain classifier. The shared encoder extracts phase-invariant anatomical features, whereas the two decoders independently model ART- and PV-specific enhancement. The Qwen3-derived prompt vectors provide phase-aware initialization and subsequently adapt through prompt–feature interaction at the bottleneck. Experiments on a single-center dataset of 86 patients show improved whole-image and lesion-focused PSNR, SSIM, MSE, and PCC relative to Pix2pix and MedGAN. Controlled ablation experiments indicate the benefits of Qwen3-based prompt initialization, subsequent prompt adaptation, and correct prompt–decoder correspondence; a reduced baseline additionally assesses the combined removal of LLTP and ART/PV domain classification. In a downstream slice-level four-class focal liver lesion classification experiment, synthetic multiphase input improved accuracy from 71.65% with NCCT alone to 85.04%, compared with 91.34% for acquired multiphase CT. These findings provide a proof of concept for LLM-guided multi-phase CT synthesis, although external validation, clinically oriented safety assessment, and reader studies remain necessary before clinical use for decision support or diagnostic triage.

## 1. Introduction

Accurate characterization of focal liver lesions (FLLs) is important for treatment planning and prognosis assessment. Contrast-enhanced computed tomography (CECT) is widely used because enhancement patterns in the arterial (ART) and portal-venous (PV) phases provide information about tumor perfusion, vascular morphology, and lesion behavior. Nevertheless, CECT requires iodinated contrast administration, which may be unsuitable for patients with renal impairment, iodine allergy, or other contraindications. Non-contrast CT (NCCT) avoids contrast-related risks but lacks the phase-specific enhancement information available in multiphase CECT. Recent clinical feasibility studies have reported that adding synthetic contrast-enhanced views to NCCT can improve diagnostic confidence or lesion detection in selected settings, while also documenting synthesis artifacts and emphasizing that synthetic images should not replace acquired CECT [1,2].

Accordingly, this work considers a complementary rather than substitutive clinical role. After appropriate external and clinical validation, synthesized phase-specific images could support preliminary clinical assessment or diagnostic triage by helping clinicians identify suspicious cases that warrant further work-up with acquired multiphase CECT. Clinicians should not use synthesized images independently to confirm or exclude disease; acquired clinical images remain essential for definitive diagnosis, model validation, and clinical decision-making.

Deep learning-based CECT synthesis has progressed from general paired image translation toward contrast-specific and multi-phase modeling. Liu et al. synthesized abdominal and pelvic CECT directly from NCCT [3], whereas Pang et al. investigated self-supervised and registered-data strategies for bidirectional NCCT–CECT translation and downstream pulmonary-vessel segmentation [4]. Uhm et al. proposed a unified three-dimensional framework that recovers missing phases while supporting kidney-cancer classification [5]. More recent CECT-specific models, including MCGAN [6], IFGAN [7], and a conditional diffusion approach [8], further illustrate the value of task-specific architectures. However, these methods generally focus on one target phase, one anatomical application, or missing-phase completion from partially available multiphase inputs rather than simultaneous ART and PV synthesis from a single NCCT image.

Medical image translation has also adopted diffusion and transformer architectures. Adversarial diffusion models can improve distribution modeling in unpaired medical image translation [9], and target-guided diffusion supports unpaired cross-modality translation [10]. Comprehensive reviews identify diffusion models as promising alternatives to GANs while noting their substantial computational requirements and the continuing need to establish anatomical fidelity and clinical reliability [11]. Meanwhile, hierarchical vision transformers provide long-range contextual modeling that can preserve shared anatomy across target phases.

A remaining challenge is to distinguish ART- and PV-specific enhancement while retaining a common anatomical representation. Completely separate models cannot exploit shared anatomical features efficiently, whereas a single undifferentiated decoder may mix phase characteristics. In addition, most existing approaches do not explicitly encode language-derived phase semantics. To address these limitations, we propose a task-prompt-guided shared-encoder dual-decoder framework for simultaneous ART and PV synthesis from NCCT. A two-dimensional Swin UNETR-based generator uses one encoder to learn phase-invariant anatomy and two independent decoders to model phase-specific contrast characteristics. Qwen3-8B independently encodes two phase descriptions to initialize learnable task prompts, and bottleneck cross-attention injects each prompt into its corresponding decoder branch. An independent ART/PV domain classifier complements two phase-specific adversarial discriminators and provides explicit phase-domain supervision. The principal contributions are as follows:

- We propose a shared-encoder dual-decoder framework that simultaneously synthesizes ART and PV images from one NCCT input while maintaining a common anatomical representation.
- We introduce independently initialized and optimized LLM-guided task prompts that provide phase-specific semantic priors for bottleneck feature modulation.
- We develop a mask-free training framework that does not require liver- or tumor-mask supervision for image generation.
- We combine two phase-specific adversarial discriminators with an independent ART/PV domain classifier and evaluate the framework through whole-image analysis, lesion-focused analysis, phase-swapped prompt control, and downstream FLL classification.

## 2. Related Work

### 2.1. Contrast-Enhanced and Multi-Phase CT Synthesis

Early paired medical image translation frameworks such as Pix2pix [12] and MedGAN [13] established adversarial and reconstruction-based mappings between medical imaging domains. More recent studies have focused specifically on synthetic contrast enhancement. Kim et al. evaluated synthetic abdominal CECT as an adjunct to NCCT in emergency-department patients and reported improvements in diagnostic confidence for several readers [1]. Liu et al. synthesized arterial, portal, and delayed abdominal/pelvic phases from NCCT [3], whereas Pang et al. combined self-supervised pretraining and registered paired data for NCCT–CECT translation and pulmonary-vessel segmentation [4]. Han et al. subsequently conducted multi-institutional clinical validation of weakly supervised synthetic abdominal CECT, showing potential benefits for lesion detection but also reporting pseudo-enhancement and vascular non-enhancement artifacts [2].

Researchers have also formulated multi-phase synthesis as a missing-modality problem. Uhm et al. jointly reconstructed missing kidney CT phases and classified tumor subtypes using a three-dimensional multi-task framework [5]. CECT-specific GAN and diffusion models have further introduced progressive generation, frequency-domain constraints, organ-aware guidance, and conditional denoising [6,7,8,14]. Collectively, these studies indicate the potential of synthetic enhancement, but most generate a single target phase, require partially observed multiphase input, use separate target-specific models, or rely on additional regional supervision. The present work instead generates ART and PV concurrently from one NCCT input using shared anatomical encoding and independent phase-specific decoding.

### 2.2. Structure-Preserving, Transformer, and Diffusion-Based Translation

Structure-preserving synthesis aims to reduce anatomically implausible changes. SA-GAN [15] combines global and local streams to preserve organ structures during MRI-to-CT translation, while TarGAN [16] jointly models global and target-region mappings. Transformer architectures provide a complementary mechanism for long-range representation learning. Swin Transformer [17] introduces hierarchical shifted-window self-attention, and Swin UNETR [18] integrates a Swin encoder with U-shaped multi-scale decoding. Prior dual-encoder research has also shown that combining local convolutional and global transformer features can improve medical image analysis [19], while DaViT [20] demonstrates the complementary roles of spatial and channel attention.

Diffusion models have emerged as alternatives to one-step adversarial translation. SynDiff combines conditional diffusion with adversarial projections and cycle consistency for unpaired medical image translation [9], while TGDM uses target guidance to reduce source-modality remnants during cross-modality synthesis [10]. A recent survey summarizes their broad use in medical generation, translation, reconstruction, and related tasks, while highlighting computational cost and clinical-validation challenges [11]. These developments motivate the use of globally contextual representations, but they do not directly address efficient simultaneous generation of two liver CECT phases from a single NCCT input.

### 2.3. Prompt Learning and Phase-Aware Generation

Prompt learning provides a mechanism for adapting pretrained semantic representations to downstream tasks. CoOp replaces manually designed context words with learnable prompt vectors [21], and MedCLIP learns medical image–text representations from unpaired biomedical images and reports [22]. Surveys of medical LLMs further emphasize the expanding role of language-derived representations in multimodal healthcare systems [23]. However, most prompt-based methods focus on classification, retrieval, or vision–language alignment rather than paired medical image synthesis.

ART and PV images contain the same underlying anatomy but differ in vascular and parenchymal enhancement. A single undifferentiated generation pathway may therefore mix phase characteristics, whereas completely separate models fail to exploit shared anatomy. The proposed framework addresses this gap by combining a shared encoder, two target-specific decoders, independently initialized Qwen3 task prompts, and phase-classification supervision. This architecture separates common anatomical representation learning from phase-aware synthesis without requiring mask supervision during training.

## 3. Method

### 3.1. Overview

The proposed framework synthesizes arterial-phase (ART) and portal-venous-phase (PV) CT images from a single non-contrast CT (NCCT) input. The network comprises a shared-encoder dual-decoder generator, two phase-specific conditional adversarial discriminators, and an independent ART/PV domain classifier. The shared encoder extracts anatomical features common to the contrast phases and passes these representations to two independent decoder branches, which generate the ART and PV images.

We introduce learnable task prompts to enhance task awareness during phase-specific generation. Unlike conventional prompt-learning strategies that use random initialization, our method initializes the prompts with semantic priors from the Qwen3-8B large language model. We design separate descriptions for the ART and PV modalities, encode them with Qwen3-8B, and use the resulting language representations to initialize the corresponding prompts. This procedure supplies phase-specific prior information before training and provides an informative starting point for subsequent prompt adaptation.

**Figure 1:**
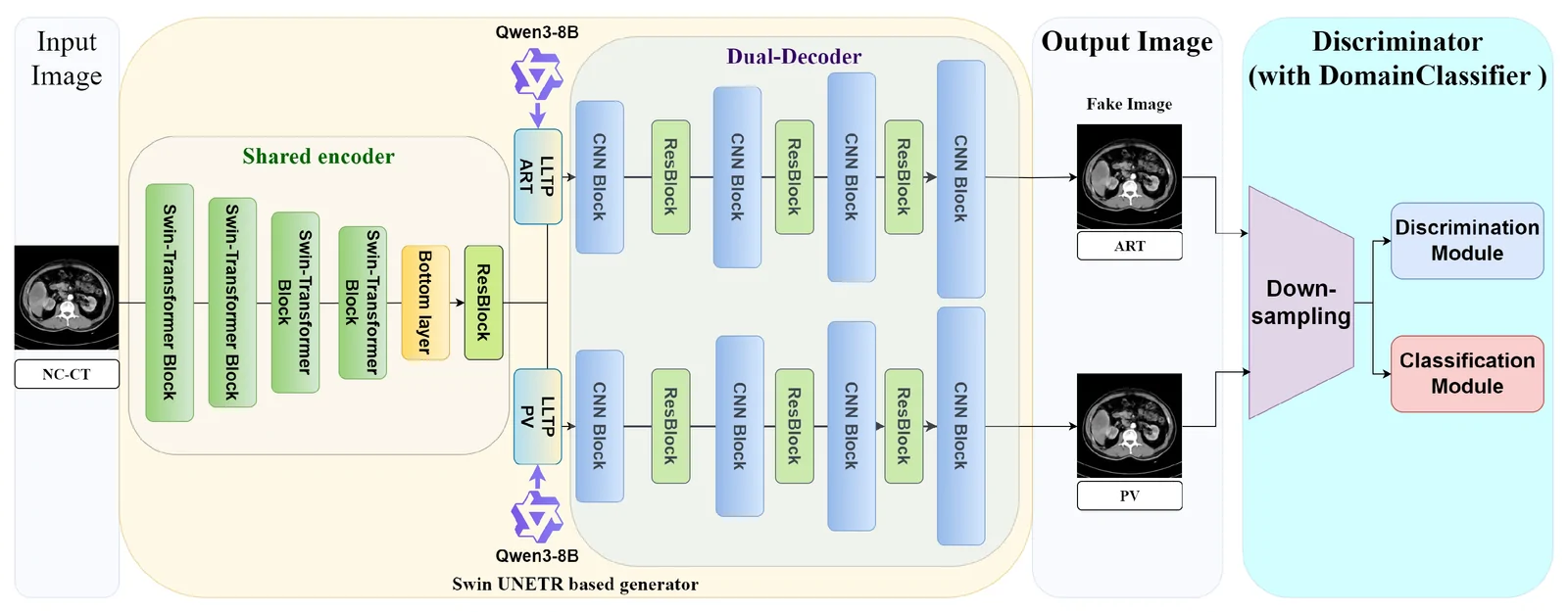
Overall architecture of the proposed shared-encoder dual-decoder Swin UNETR-based GAN framework. A shared Swin Transformer-based encoder extracts anatomical features from NCCT, and two independent decoding branches generate arterial-phase (ART) and portal-venous-phase (PV) CT images. Two separate conditional adversarial discriminators evaluate ART and PV realism, while an additional independent domain classifier distinguishes ART from PV.

Each phase-specific adversarial discriminator evaluates its corresponding synthesized output and provides real/fake supervision. In parallel, the independent domain classifier predicts whether a real or synthesized contrast-enhanced image belongs to the ART or PV domain. The domain classifier does not share an output head with either adversarial discriminator. Together, these supervision networks encourage image realism and phase-specific contrast characteristics. Figure 1 illustrates the overall architecture.

### 3.2. Shared Encoder and Dual-Decoder Generator

The generator is a two-dimensional adaptation of Swin UNETR designed for slice-based CT synthesis. Given an NCCT input $I_{\mathrm{NC}}$, the shared Swin Transformer encoder extracts hierarchical representations $\left\{E_{1},E_{2},E_{3},E_{4},E_{5}\right\}$ containing local anatomical detail and long-range contextual information. Because NCCT, ART, and PV images from the same subject share anatomy, the encoder is common to both synthesis tasks and provides a unified anatomical feature space.

Two independent decoder branches reconstruct ART and PV images from the shared representations. Each decoder progressively upsamples its bottleneck representation and fuses it with corresponding encoder features through skip connections. The ART decoder specializes in arterial enhancement, whereas the PV decoder specializes in portal-venous enhancement. The synthesis mapping is written as

(1) $${\hat{I}}_{\mathrm{ART}}={\mathrm{Dec}}_{\mathrm{ART}}\;\left(E\left(I_{\mathrm{NC}}\right);p_{\mathrm{ART}}\right),\;{\hat{I}}_{\mathrm{PV}}={\mathrm{Dec}}_{\mathrm{PV}}\;\left(E\left(I_{\mathrm{NC}}\right);p_{\mathrm{PV}}\right),$$

where E(·) denotes the shared encoder, ${\mathrm{Dec}}_{\mathrm{ART}}\left(\cdot \right)$ and ${\mathrm{Dec}}_{\mathrm{PV}}\left(\cdot \right)$ denote the phase-specific decoders, and $p_{\mathrm{ART}}$ and $p_{\mathrm{PV}}$ are the corresponding learnable task prompts. This design preserves anatomical consistency through shared encoding while allowing independent phase-specific feature modulation and reconstruction.

### 3.3. LLM-Guided Learnable Task Prompt Initialization

The LLTP mechanism provides an informative phase-specific initialization instead of starting both task embeddings from random values. We use two text descriptions: “Describe the brightness pattern of arterial phase CT images after contrast injection, where arteries and hypervascular regions show strong enhancement” for ART, and “Describe the brightness pattern of portal venous phase CT images after contrast injection, where the liver parenchyma and portal veins appear brighter and more homogeneous” for PV. The Qwen3-8B tokenizer and chat template process each description independently.

For each phase t∈{ART,PV}, we extract the final hidden state of the last input token from the last Qwen3-8B layer:

(2) $$e_{t}=H_{t}^{\left(L\right)}\left[-1\right]\in R^{4096}.$$

**Figure 2:**
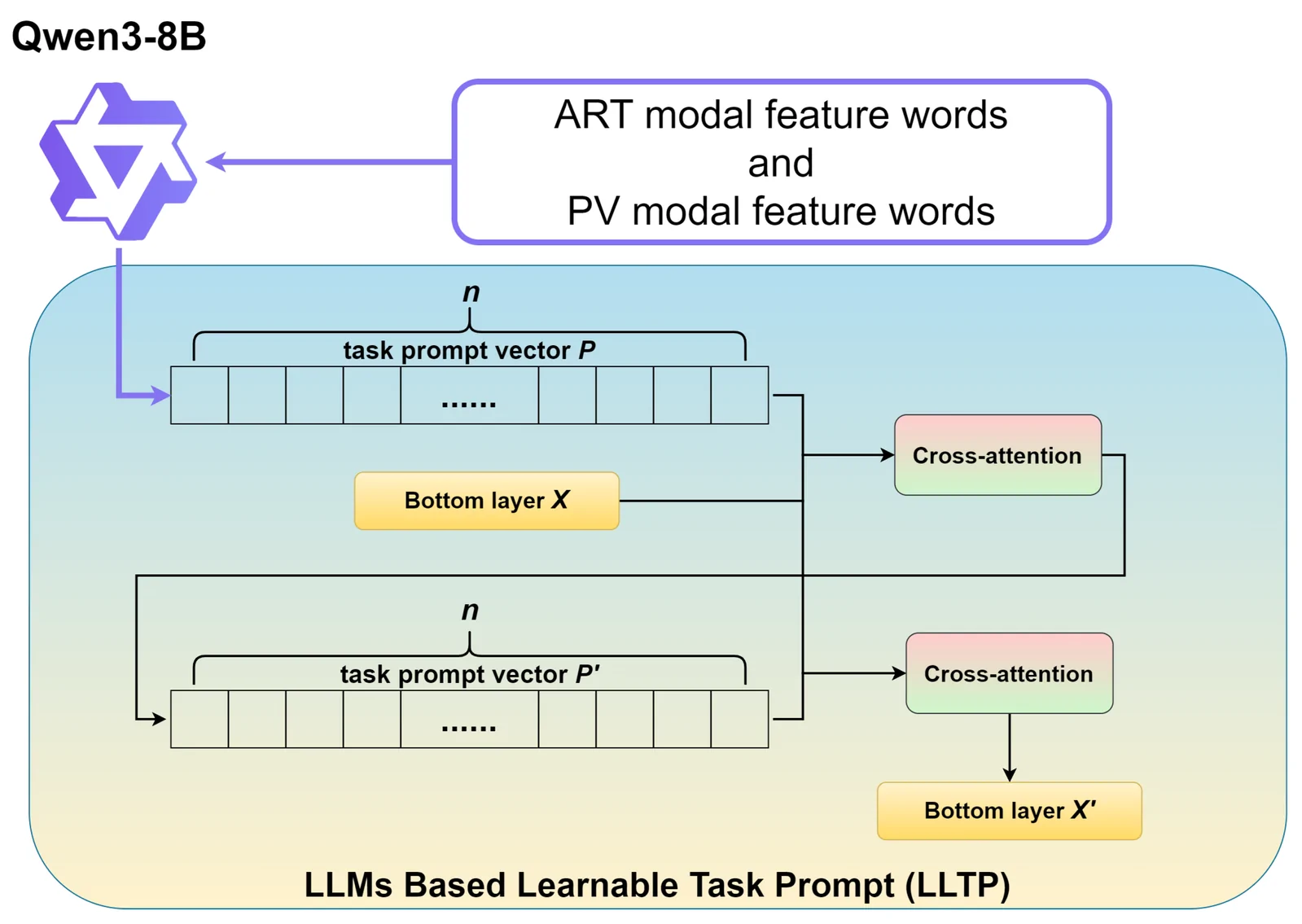
Workflow of the LLM-guided learnable task prompt (LLTP) mechanism. Qwen3-8B encodes separate ART and PV text descriptions, and the L2-normalized 4096-dimensional representations initialize two independent task embeddings. Dual-stage cross-attention injects each embedding only into its corresponding decoder branch at the bottleneck. Training keeps the task embeddings fixed during the first five epochs and optimizes them jointly with the generator thereafter.

We store the extracted vectors as 32-bit floating-point arrays and apply L2 normalization before initializing the task bank:

(3) $$p_{t}^{\left(0\right)}=\frac{e_{t}}{{∥e_{t}∥}_{2}+\epsilon }.$$

where ϵ>0 is a small constant used to avoid division by zero. Because both the Qwen3 representation and task embedding have 4096 dimensions, the initialization requires no additional projection. We copy the normalized vectors directly into the ART and PV entries of the learnable embedding bank and maintain the two embeddings independently during injection and optimization. Figure 2 summarizes the LLTP initialization and the subsequent prompt–feature interaction workflow.

Dual-stage cross-attention performs prompt–feature interaction at the bottleneck of each decoder branch. Let $X_{t}$ denote the bottleneck feature tokens of branch *t* and let $p_{t}$ denote its task embedding. In the first stage, feature tokens query a key–value pair derived from the task embedding and an additional learnable residual vector r:

(4) $$\begin{array}{cccc} Q_{t}^{\left(1\right)} & =W_{q}^{\left(1\right)}X_{t}, & \left[K_{t}^{\left(1\right)},V_{t}^{\left(1\right)}\right] & =W_{kv}^{\left(1\right)}\left(p_{t}+r\right),\\ Z_{t} & =X_{t}+\mathrm{Attn}\left(Q_{t}^{\left(1\right)},K_{t}^{\left(1\right)},V_{t}^{\left(1\right)}\right). & & \end{array}$$

A residual multilayer transformation then stabilizes the updated feature representation. In the second stage, the prompt queries globally pooled key and value representations derived from $Z_{t}$:

(5) $$\begin{array}{cc} q_{t}^{\left(2\right)} & =W_{q}^{\left(2\right)}p_{t},\\ k_{t}^{\left(2\right)} & =\mathrm{GAP}\left(W_{k}^{\left(2\right)}Z_{t}\right),\;v_{t}^{\left(2\right)}=\mathrm{GAP}\left(W_{v}^{\left(2\right)}Z_{t}\right),\\ u_{t} & =\mathrm{Attn}(q_{t}^{\left(2\right)},k_{t}^{\left(2\right)},v_{t}^{\left(2\right)}). \end{array}$$

The mechanism converts the resulting task-conditioned vector into channel-wise scale and shift parameters that modulate the bottleneck features through a residual FiLM-style operation:

(6) $${\tilde{X}}_{t}=Z_{t}+W_{o}\;\left(\mathrm{GN}\left(Z_{t}\right)⊙\left[1+\gamma \left(u_{t}\right)\right]+\beta \left(u_{t}\right)\right).$$

This bidirectional interaction allows phase semantics to modify image features while adapting the semantic representation to the visual context. During training, we freeze the task-bank embeddings for the first five epochs and optimize them jointly with the generator from the sixth epoch onward; the prompt–feature fusion layers remain trainable throughout. We also apply prompt dropout to reduce over-reliance on semantic conditioning.

The full model and random-initialization ablation differ only in task-embedding initialization: both variants use the same embedding bank, cross-attention architecture, freezing schedule, and subsequent optimization, whereas only the full model starts from Qwen3-derived phase semantics.

### 3.4. Phase-Specific Adversarial Discriminators and Domain Classifier

The framework uses three independent supervision networks rather than one discriminator with two output heads. Two conditional adversarial discriminators, $D_{\mathrm{ART}}$ and $D_{\mathrm{PV}}$, separately evaluate the realism of the synthesized ART and PV images. Each phase-specific discriminator receives the NCCT image together with either the corresponding acquired target image or the synthesized output and produces a real/fake logit.

We implement the additional domain classifier, $C_{\mathrm{dom}}$, as a separate network. Given the NCCT image together with an ART or PV image, the classifier predicts one of two phase labels. During the domain-classifier update, acquired ART and PV images provide phase-labeled supervision. During the generator update, we hold the classifier parameters fixed and use its predictions on synthesized ART and PV images to provide phase-aware gradients to the generator. Thus, separate networks and objectives govern adversarial realism and ART/PV domain discrimination.

This separation is important because ART and PV images share highly similar anatomical structures while differing primarily in enhancement patterns and intensity distributions. The two adversarial discriminators encourage each output to match its target-phase image distribution, whereas the independent domain classifier explicitly penalizes phase ambiguity. Together, they promote realistic synthesis while preserving the intended phase-specific characteristics.

### 3.5. Loss Function

We optimize the framework using reconstruction, phase-specific adversarial, and domain-classification objectives. The $L_{1}$ reconstruction loss penalizes deviations between each synthesized image and its patient-specific acquired target:

(7) $$L_{L1}=L_{L1}^{\mathrm{ART}}+L_{L1}^{\mathrm{PV}},$$

where

(8) $$L_{L1}^{\mathrm{ART}}={∥I_{\mathrm{ART}}^{*}-{\hat{I}}_{\mathrm{ART}}∥}_{1},\;L_{L1}^{\mathrm{PV}}={∥I_{\mathrm{PV}}^{*}-{\hat{I}}_{\mathrm{PV}}∥}_{1}.$$

For each target phase t∈{ART,PV}, the discriminator-side adversarial loss is

(9) $$\begin{array}{cc} L_{\mathrm{adv},D}^{t}=\frac{1}{2}[ & \mathrm{BCE}(D_{t}\left(I_{\mathrm{NC}},I_{t}^{*}\right),1)\\ & +\mathrm{BCE}(D_{t}\left(I_{\mathrm{NC}},{\hat{I}}_{t}\right),0)], \end{array}$$

and the corresponding generator-side adversarial loss is

(10) $$L_{\mathrm{adv},G}^{t}=\mathrm{BCE}\left(D_{t}\left(I_{\mathrm{NC}},{\hat{I}}_{t}\right),1\right).$$

Using phase label 0 for ART and 1 for PV, we train the independent domain classifier on acquired images according to

(11) $$\begin{array}{cc} L_{\mathrm{cls}}^{\mathrm{dom}}=\frac{1}{2}[ & \mathrm{CE}(C_{\mathrm{dom}}\left(I_{\mathrm{NC}},I_{\mathrm{ART}}^{*}\right),0)\\ \; & +\mathrm{CE}(C_{\mathrm{dom}}\left(I_{\mathrm{NC}},I_{\mathrm{PV}}^{*}\right),1)]. \end{array}$$

The generator-side domain-classification loss is

(12) $$\begin{array}{cc} L_{\mathrm{cls}}^{G}=\frac{1}{2}[ & \mathrm{CE}(C_{\mathrm{dom}}\left(I_{\mathrm{NC}},{\hat{I}}_{\mathrm{ART}}\right),0)\\ \; & +\mathrm{CE}(C_{\mathrm{dom}}\left(I_{\mathrm{NC}},{\hat{I}}_{\mathrm{PV}}\right),1)], \end{array}$$

which encourages the domain classifier to recognize each synthesized output as its intended phase.

The overall generator objective is

(13) $$\begin{array}{cc} L_{G}= & \lambda_{\mathrm{adv}}^{\mathrm{ART}}L_{\mathrm{adv},G}^{\mathrm{ART}}+\lambda_{\mathrm{adv}}^{\mathrm{PV}}L_{\mathrm{adv},G}^{\mathrm{PV}}\\ \; & +\lambda_{L1}L_{L1}+\lambda_{\mathrm{cls}}^{G}L_{\mathrm{cls}}^{G}. \end{array}$$

For compactness, the total supervision-network objective can be written as

(14) $$L_{\sup}=L_{\mathrm{adv},D}^{\mathrm{ART}}+L_{\mathrm{adv},D}^{\mathrm{PV}}+\lambda_{\mathrm{cls}}^{\mathrm{dom}}L_{\mathrm{cls}}^{\mathrm{dom}},$$

although $D_{\mathrm{ART}}$, $D_{\mathrm{PV}}$, and $C_{\mathrm{dom}}$ are independent networks with separate optimizers. We set $\lambda_{\mathrm{adv}}^{\mathrm{ART}}=10.0$, $\lambda_{\mathrm{adv}}^{\mathrm{PV}}=15.0$, $\lambda_{L1}=100$, $\lambda_{\mathrm{cls}}^{\mathrm{dom}}=1.0$, and $\lambda_{\mathrm{cls}}^{G}=1.5$. We set all pairwise difference-image adversarial weights (ART–PV, ART–NC, and PV–NC) and the corresponding difference-reconstruction weights to zero in the reported experiments. Consequently, these terms do not contribute to generator optimization, and the effective objective above omits them.

## 4. Experiments and Results

### 4.1. Dataset

We used a single-center multiphase CT dataset collected at Sir Run Run Shaw Hospital between 2015 and 2017. The dataset contains multidetector helical CT images with a slice thickness of 5–7 mm and an original in-plane resolution of 512×512 pixels. Each patient had NC, ART, and PV scans together with tumor masks, liver masks, and focal liver lesion labels. The cohort comprised 86 patients with hepatocellular carcinoma (HCC), focal nodular hyperplasia (FNH), metastases (METS), or hepatic cysts and included no healthy controls.

According to the retained data-partition records, the original data-preparation team partitioned the data strictly at the patient level before providing the curated and de-identified slice dataset for the present study. The training set contained 69 patients (16 with FNH, 18 with HCC, 26 with hepatic cysts, and 9 with METS), and the test set contained 17 patients (4 with FNH, 4 with HCC, 6 with hepatic cysts, and 3 with METS). No patient contributed slices to both partitions. The resulting sets contained 774 matched NC–ART–PV slice triplets for training and 127 matched triplets for testing. Although the patient-level separation between the two partitions was preserved, the de-identified analysis dataset did not retain identifiers linking individual slices within each partition to their source patients. Consequently, the 127 test slices could not be retrospectively regrouped into the corresponding 17 patients for patient-level metric aggregation, confidence-interval estimation, or paired statistical testing.

The dataset available for the present study consisted of a curated and previously preprocessed collection of paired slices rather than raw multiphase CT volumes. Available records indicate that the team performed lesion-based inter-phase slice matching before this study. For each patient, the team first selected the NC, ART, and PV slices containing the largest tumor cross-sectional area and then added the remaining tumor-containing slices sequentially according to lesion location and anatomical correspondence. We used these curated triplets directly and performed no additional geometric registration. Because the retained records contain neither transformation parameters (rigid, affine, or deformable) nor quantitative residual-misalignment measurements, we refer to this preprocessing as slice matching rather than geometric image registration.

We resized all images to 256×256 pixels, applied a soft-tissue window with a level of 40 HU and a width of 400 HU, and rescaled the intensities to [0,255]. Before network input, we further normalized the resulting 8-bit images to [−1,1].

### 4.2. Implementation Details

Table 1 summarizes the implementation settings. We initialized the Swin Transformer encoder with officially released pretrained weights and subsequently fine-tuned it jointly with the task-specific components. In each iteration, we alternated one generator update with one update of each active phase-specific adversarial discriminator and one update of the independent domain classifier.

### 4.3. Evaluation Metrics

We evaluated the generated ART and PV images using mean squared error (MSE), peak signal-to-noise ratio (PSNR), structural similarity index (SSIM), and Pearson correlation coefficient (PCC). Lower MSE values indicate smaller reconstruction errors, whereas higher PSNR, SSIM, and PCC values indicate better agreement with the corresponding acquired phase.

For a synthesized image *x* and its ground-truth image *y*, MSE is defined as

(15) $$\mathrm{MSE}\left(x,y\right)=\frac{1}{N}\sum_{i=1}^{N}{\left(x_{i}-y_{i}\right)}^{2},$$

where *N* is the number of evaluated pixels. PSNR is defined as

(16) $$\mathrm{PSNR}\left(x,y\right)=10\log_{10}\;\left(\frac{{\mathrm{MAX}}_{I}^{2}}{\mathrm{MSE}(x,y)}\right),$$

where ${\mathrm{MAX}}_{I}=255$ for the evaluated 8-bit grayscale images.

SSIM evaluates luminance, contrast, and structural agreement:

(17) $$\mathrm{SSIM}\left(x,y\right)=\frac{\left(2\mu_{x}\mu_{y}+C_{1}\right)\left(2\sigma_{xy}+C_{2}\right)}{\left(\mu_{x}^{2}+\mu_{y}^{2}+C_{1}\right)\left(\sigma_{x}^{2}+\sigma_{y}^{2}+C_{2}\right)},$$

where $\mu_{x}$ and $\mu_{y}$ are mean intensities, $\sigma_{x}^{2}$ and $\sigma_{y}^{2}$ are variances, and $\sigma_{xy}$ is covariance. The stabilization constants were

(18) $$C_{1}={\left(K_{1}L\right)}^{2}=6.5025,\;C_{2}={\left(K_{2}L\right)}^{2}=58.5225,$$

with $K_{1}=0.01$, $K_{2}=0.03$, and dynamic range L=255.

PCC measures the linear correlation between corresponding intensity values:

(19) $$\mathrm{PCC}\left(x,y\right)=\frac{\sum_{i=1}^{N}\left(x_{i}-\bar{x}\right)\left(y_{i}-\bar{y}\right)}{\sqrt{\sum_{i=1}^{N}{\left(x_{i}-\bar{x}\right)}^{2}}\sqrt{\sum_{i=1}^{N}{\left(y_{i}-\bar{y}\right)}^{2}}},$$

where $\bar{x}$ and $\bar{y}$ denote the corresponding mean intensities.

We computed whole-image metrics over the complete grayscale images. For lesion-focused evaluation, the provided tumor masks defined the pixels for MSE, PSNR, and PCC. We computed SSIM on the smallest rectangular crop enclosing each lesion mask because SSIM evaluates local neighborhoods. We then averaged the slice-wise values over the test set for each synthesis task.

### 4.4. Downstream FLL Classification

To examine whether the synthesized phases contain task-relevant information beyond pixel-level similarity, we conducted a slice-level FLL classification experiment with four classes: cyst, FNH, HCC, and METS. We trained two ImageNet-pretrained ResNet-50 classifiers. The NC-only classifier received the NC image replicated across three input channels, [NC,NC,NC], whereas the multiphase classifier received only acquired triplets, [NC,ART,PV], during training. We did not use synthetic images to train either classifier. At test time, we applied the same trained multiphase classifier to $\left[\mathrm{NC},\hat{\mathrm{ART}},\hat{\mathrm{PV}}\right]$ and [NC,ART,PV]. This design enables a direct comparison between synthetic and acquired multiphase inputs under identical classifier parameters.

We resized each complete CT slice to 224×224 pixels without tumor masks, lesion crops, or other region-of-interest preprocessing and normalized the three input channels using the ImageNet mean and standard deviation. We fixed the ResNet-50 convolutional backbone and BatchNorm statistics, replaced the final fully connected layer, and optimized only this layer for four-class prediction. We trained both classifiers for 100 epochs using cross-entropy loss, Adam with a learning rate of $1\times 10^{-4}$, a batch size of 10, and a random seed of 42. We did not use validation or test results for checkpoint selection. Both experiments used the same 774 training slices and 127 test slices from the patient-level split. We measured performance using accuracy, macro-averaged F1 score, and per-class recall.

### 4.5. Experimental Results and Analysis

Table 2 and Table 3 compare Pix2pix [12], MedGAN [13], and the proposed method on the NC→ART and NC→PV synthesis tasks, respectively. We report whole-image and lesion-area PSNR, SSIM, MSE, and PCC. The whole-image metrics characterize overall reconstruction quality and global anatomical consistency, whereas the lesion-area metrics focus on focal liver lesion (FLL) regions of greater clinical relevance.

For the NC→ART synthesis task, the proposed method achieves the best result for every whole-image and lesion-area metric. At the whole-image level, it attains the highest PSNR (32.96), SSIM (0.7972), and PCC (0.9529) and the lowest MSE (33.38). In the lesion areas, it also attains the highest PSNR (29.22), SSIM (0.4284), and PCC (0.4358) and the lowest MSE (79.55). Relative to Pix2pix and MedGAN, these results indicate better reconstruction fidelity and closer preservation of local phase-specific enhancement patterns within the evaluated FLL regions.

For the NC→PV synthesis task, the proposed method again achieves the best result for every metric. It obtains the highest whole-image PSNR (32.84), SSIM (0.7870), and PCC (0.9541) and the lowest whole-image MSE (34.45). In the lesion areas, it achieves the highest PSNR (29.11), SSIM (0.4086), and PCC (0.4979) and the lowest MSE (80.55). These improvements over Pix2pix and MedGAN indicate more accurate reconstruction of portal-venous-phase contrast distributions in both global anatomy and the evaluated lesion regions.

Table 4 and Table 5 report five ablation variants: (1) a reduced model without LLTP or the domain classifier; (2) a model with two independently and randomly initialized task prompts, which remain fixed for five epochs and undergo optimization from the sixth epoch onward; (3) a model with correctly assigned Qwen3-initialized prompts that remain fixed throughout training; (4) a phase-swapped control that assigns the PV prompt to the ART branch and the ART prompt to the PV branch while keeping both prompts fixed; and (5) the full model, which applies the same five-epoch freezing and subsequent optimization schedule to correctly assigned Qwen3-initialized prompts. All four prompt-containing variants retain the domain classifier. The random-initialization and full-model comparison changes only the prompt initialization source. The correctly assigned frozen-prompt and full-model comparison evaluates prompt adaptation, whereas the correctly assigned and phase-swapped frozen-prompt comparison evaluates prompt–decoder correspondence. Because the reduced baseline removes LLTP and the domain classifier simultaneously, comparisons with this baseline reflect their combined contribution and cannot isolate either component.

For the NC→ART task, the full model achieves the best value for every reported metric. Relative to the reduced model without LLTP and the domain classifier, the randomly initialized and subsequently adapted prompt variant improves seven of the eight metrics, with whole-image PCC being the exception. This comparison represents the joint addition of the prompt mechanism and domain classifier and therefore does not isolate the prompt effect. More directly, the full model and random-initialization variant share the same architecture, domain classifier, freezing schedule, and prompt optimization; replacing random initialization with Qwen3-derived initialization improves all eight metrics. The full model also outperforms the correctly assigned frozen-prompt variant across all metrics, indicating that adapting the Qwen3-initialized prompts after epoch 5 is beneficial in the reported experiment. Swapping the frozen prompt assignments reduces whole-image SSIM from 0.7531 to 0.7101 and lesion-area PCC from 0.3569 to 0.2928 relative to the correctly assigned frozen prompts, while increasing whole-image and lesion-area MSE. These changes support the importance of maintaining the intended correspondence between phase-specific semantic initialization and the associated decoder branch.

The NC→PV results show a similar trend. The randomly initialized and subsequently adapted prompt variant improves all eight metrics relative to the reduced model, although this comparison again includes the domain classifier and cannot isolate the prompt contribution. In the controlled comparison that changes only prompt initialization, the full model outperforms the randomly initialized variant on every whole-image and lesion-area metric. The full model also improves every metric relative to the correctly assigned frozen-prompt variant. Relative to the correctly assigned frozen prompts, phase swapping decreases whole-image SSIM from 0.7700 to 0.6791 and lesion-area PCC from 0.4574 to 0.3086, while increasing whole-image MSE from 35.88 to 38.61 and lesion-area MSE from 83.89 to 86.69. Within these reported runs, the results consistently favor Qwen3-derived over random prompt initialization, prompt adaptation over permanently frozen prompts, and correct prompt–decoder assignments over phase-swapped assignments.

Overall, the quantitative results favor the proposed method over the two GAN-based baselines for both ART and PV synthesis. The whole-image and lesion-area improvements suggest that the task-prompt-guided dual-decoder framework preserves global anatomical structure while better capturing local phase-specific contrast characteristics within focal liver lesions. Together with the ablation study, these findings support the combination of shared anatomical encoding, LLM-guided task prompts, and phase-classification supervision for multi-phase CT synthesis from NCCT.

### 4.6. Downstream Classification Results

Table 6 summarizes the slice-level four-class FLL classification results. The NC-only classifier achieved an accuracy of 0.7165 and a macro-F1 score of 0.6997. Replacing the two duplicated NC channels with synthetic ART and PV images increased accuracy to 0.8504 and macro-F1 to 0.8534, corresponding to an absolute accuracy gain of 13.39 percentage points and a macro-F1 increase of 0.1537. The acquired multiphase input achieved the highest overall performance, with an accuracy of 0.9134 and a macro-F1 score of 0.9043.

Relative to NCCT alone, synthetic multiphase input improved FNH recall from 0.6250 to 0.9167, METS recall from 0.5417 to 0.8333, and HCC recall from 0.7333 to 0.8000; cyst recall remained at 0.8824. These results suggest that the multiphase classifier, which received only acquired multiphase images during training, can use complementary information from the synthesized phases. Nevertheless, the remaining performance gap relative to acquired multiphase CT indicates that the synthesized images do not reproduce all task-relevant contrast information.

### 4.7. Qualitative Results

**Figure 3:**
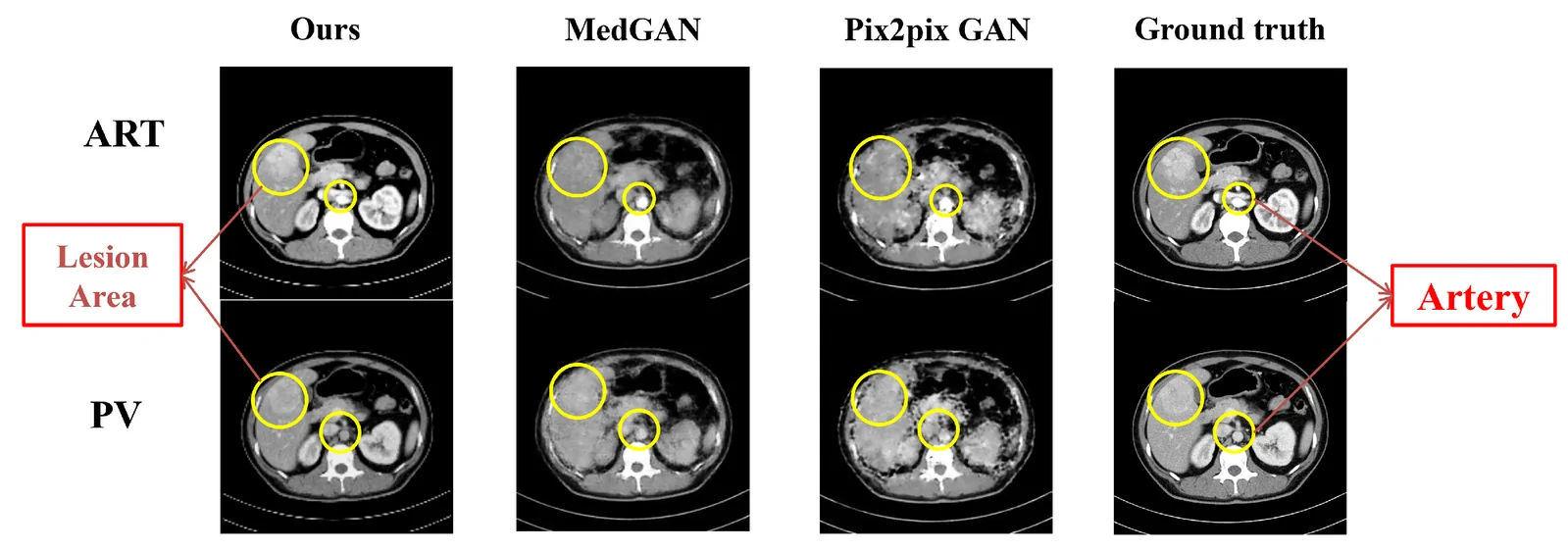
Qualitative results for NC→ART and NC→PV synthesis. Yellow circles mark the same anatomical locations across the compared methods and ground-truth images. The smaller circles highlight the aorta, whose phase-specific enhancement is an important visual cue for distinguishing ART from PV images, whereas the larger circles highlight the focal liver lesion region of interest (lesion ROI), which corresponds to the lesion mask used for lesion-focused quantitative evaluation. The circles direct the reader’s attention to these key regions.

Figure 3 compares Pix2pix [12], MedGAN [13], and the proposed method on the NC→ART and NC→PV synthesis tasks; the corresponding acquired contrast-enhanced CT images serve as ground truth. Yellow circles indicate the same anatomical locations for every method. The smaller circles highlight the aorta, whose enhancement provides an important cue for distinguishing ART from PV images, and the larger circles highlight the focal liver lesion ROI that defines the mask for lesion-focused quantitative evaluation.

Figure 3 shows that Pix2pix produces diffuse appearances and unclear structural boundaries, especially in lesion regions and around vascular structures. Although its overall enhancement follows the target phase, its synthesized images exhibit unstable local intensity patterns and poorly defined anatomical contours. MedGAN produces more realistic textures but retains inconsistencies in local enhancement distribution and blurred boundaries within the highlighted regions. Its lesion appearance and surrounding contrast transitions also remain less coherent than those in the ground truth.

In contrast, the proposed framework produces more anatomically consistent ART and PV appearances. Within the circled regions, its outputs show closer qualitative agreement with the reference images in lesion boundaries, aortic enhancement, and phase-specific enhancement patterns. The proposed method also provides clearer lesion depiction and more coherent enhancement distributions within focal liver lesions while maintaining global structural integrity. These observations are consistent with the quantitative comparisons.

## 5. Discussion

The quantitative and qualitative results indicate that separating common anatomical encoding from phase-specific decoding is beneficial for simultaneous ART and PV synthesis. The two decoders share the same NCCT-derived anatomical representation but receive independent semantic task prompts; this design aims to reduce mixing between arterial and portal-venous characteristics. In the controlled initialization comparison, Qwen3-initialized prompts outperform randomly initialized prompts even though both variants use the same domain classifier, prompt-fusion architecture, five-epoch freezing period, and subsequent prompt optimization. Likewise, the comparison with permanently frozen Qwen3 prompts favors subsequent prompt adaptation. The reduced baseline removes both LLTP and the domain classifier and therefore provides evidence only about their combined inclusion, not the isolated effect of either component. Moreover, swapping the ART and PV prompt assignments while freezing both prompts consistently degrades synthesis performance, particularly for PV whole-image SSIM and lesion-area PCC. This negative control supports the importance of phase-specific prompt–decoder correspondence, although it does not by itself prove that the language model encodes complete clinical knowledge of enhancement physiology. These findings suggest that the LLM acts as an initialization source for task semantics rather than directly generating medical images or textual diagnoses.

Unlike region-aware methods such as OA-GAN [14], the proposed framework requires no liver or tumor masks during synthesis training and produces ART and PV outputs within a single model. The independent ART/PV domain classifier provides explicit phase supervision that complements pixel reconstruction and the two phase-specific adversarial objectives. Nevertheless, the present study compares the framework only with Pix2pix and MedGAN. Future studies should include recent GAN- and diffusion-based methods to establish its position relative to the current state of the art.

The reference ART and PV images in this study are patient-specific acquired scans rather than healthy anatomical templates. All 86 patients had focal liver lesions, including HCC, FNH, METS, or cysts; consequently, the evaluation includes clinically meaningful abnormalities. However, similarity to a paired reference does not establish the medical correctness of every synthesized abnormality. Pixel-level metrics may fail to detect false enhancement, obscured lesions, altered vascular findings, or subtle changes in enhancement patterns. In a future clinically validated workflow, synthesized phase-specific views could support preliminary assessment or diagnostic triage by flagging suspicious cases and informing the decision to perform definitive multiphase CECT. Clinicians must not use these images as substitutes for acquired CECT or as independent evidence to confirm or exclude disease. This cautious positioning is consistent with clinical studies that used synthetic CECT as an adjunct to NCCT and reported both potential reader benefits and clinically relevant artifacts [1,2].

The downstream classification experiment provides preliminary task-oriented evidence beyond image-similarity metrics. Relative to the separate NC-only classifier, the multiphase classifier increased accuracy from 71.65% to 85.04% and macro-F1 from 0.6997 to 0.8534 when it received synthetic multiphase input. We trained this classifier exclusively on acquired multiphase images and used identical parameters to test synthetic and acquired multiphase inputs. The result therefore suggests that the generated ART and PV images contain complementary phase-related information that a model trained on real multiphase data can recognize. However, acquired multiphase input remained superior overall. This slice-level experiment provides only a preliminary indication of task relevance and does not establish that synthetic CECT can replace acquired phases or support autonomous diagnosis.

This study has several limitations. First, the relatively small single-center dataset limits generalizability across scanners, acquisition protocols, institutions, and patient populations. Second, the original data-preparation team completed lesion-based inter-phase slice matching before providing the curated dataset. Because the retained records contain neither the original geometric transformation parameters nor quantitative residual-misalignment measurements, we could not retrospectively assess small respiratory or anatomical inconsistencies. Third, although the original training–test partition was performed strictly at the patient level, the de-identified slice-level dataset available for the present analysis did not retain identifiers linking individual slices within each partition to their source patients. Consequently, the 127 test slices could not be reliably regrouped into the corresponding 17 patients, which precluded patient-level metric aggregation, confidence-interval estimation, paired statistical testing, and patient-level downstream analysis. Treating individual slices as statistically independent observations would ignore within-patient correlation and could produce misleadingly narrow confidence intervals or significance estimates. The downstream classification experiment therefore remained a slice-level analysis on the same single-center cohort and used a fixed ImageNet-pretrained feature extractor. Fourth, we did not conduct a formal blinded radiologist assessment or external validation. More broadly, the evaluation omits repeated-training stability analysis, uncertainty estimation, explainability assessment, and blinded reader evaluation. These omissions are particularly important for GAN-based synthesis because hallucination, training variability, and black-box behavior remain unresolved safety concerns. Legal responsibility, patient consent, and governance of synthetic clinical images also require institution-specific prospective consideration.

Future work will prioritize multi-center external validation, stronger contemporary baselines, repeated-run stability evaluation, and clinically oriented reader studies. Future datasets will preserve anonymized slice-to-patient linkage so that synthesis metrics and downstream predictions can be aggregated at the patient level and evaluated using appropriate confidence intervals and paired statistical tests. These studies should explicitly evaluate lesion conspicuity, enhancement-pattern preservation, hallucinated or missed findings, and patient-level downstream diagnostic tasks. Additional negative-control prompt experiments and alternative language encoders may further clarify whether clinically meaningful semantic initialization accounts for the observed performance gains.

## 6. Conclusions

We proposed an LLM-guided shared-encoder dual-decoder framework that simultaneously synthesizes ART and PV images from NCCT. The model combines a pretrained Swin Transformer encoder, phase-specific decoders, independently initialized learnable task prompts, two phase-specific adversarial discriminators, and an independent ART/PV domain classifier. On a patient-level split of a single-center FLL dataset, the model achieved better whole-image and lesion-focused quantitative metrics than Pix2pix and MedGAN. The ablation study indicates benefits from semantic prompt initialization and prompt adaptation, while the phase-swapped frozen-prompt control highlights the importance of correct phase-specific prompt–decoder correspondence.

In a preliminary downstream four-class FLL classification experiment, synthetic multiphase input improved slice-level accuracy from 71.65% with NCCT alone to 85.04%, while acquired multiphase CT achieved 91.34%. These results provide a proof of concept for introducing language-derived phase semantics into multi-output medical image synthesis and suggest that the synthesized phases contain complementary task-relevant information. Rather than replacing acquired CECT, a future clinically validated system could provide complementary information for preliminary assessment and help determine whether a patient warrants further multiphase CECT work-up. Before considering such use, future studies must include larger multi-center cohorts, contemporary baseline comparisons, patient-level statistical analysis, and radiologist reader studies.

## Figures and Tables

**Table 1:** Implementation and training configuration of the proposed framework.

Setting	Configuration
Hardware	Two NVIDIA GeForce RTX 3090 GPUs (approximately 24 GB each)
Operating system	Linux 5.15.0-138-generic, x86_64
Software environment	Python 3.7.13; PyTorch 1.12.1; torchvision 0.13.1; MONAI 1.1.0
GPU libraries	CUDA 11.3; cuDNN 8.3.2
Input size and normalization	1×256×256; normalized to [−1,1]
Encoder initialization	Officially released pretrained Swin Transformer weights; jointly fine-tuned
Optimizer	Adam, $\beta_{1}=0.5$, $\beta_{2}=0.999$
Learning rate	0.005
Batch size	64
Training epochs	300
Active supervision networks	Independent ART and PV conditional adversarial discriminators plus an independent two-class domain classifier
Network update schedule	One generator update and one update of each active supervision network per iteration
ART/PV adversarial weights	$\lambda_{\mathrm{adv}}^{\mathrm{ART}}=10.0$, $\lambda_{\mathrm{adv}}^{\mathrm{PV}}=15.0$
Reconstruction weight	$\lambda_{L1}=100$
Domain-classification weights	$\lambda_{\mathrm{cls}}^{\mathrm{dom}}=1.0$, $\lambda_{\mathrm{cls}}^{G}=1.5$
Pairwise difference-image terms	ART–PV, ART–NC, and PV–NC adversarial and reconstruction weights set to 0
Task-prompt dimension	4096
Task-prompt initialization	Qwen3-8B final-layer, final-token representation; L2 normalized
Prompt fusion and location	Dual-stage cross-attention at the bottleneck
Prompt freezing schedule	Task embeddings frozen for epochs 1–5 and optimized from epoch 6
Prompt dropout	0.1
Mask supervision during training	Not used

**Table 2:** Quantitative comparison on the NC→ART synthesis task. Higher PSNR, SSIM, and PCC values indicate better performance, while lower MSE indicates better performance. Bold values indicate the best performance in each column.

Methods	Whole Image				Lesion Area			
	PSNR ↑	SSIM ↑	MSE ↓	PCC ↑	PSNR ↑	SSIM ↑	MSE ↓	PCC ↑
Pix2pix [12]	32.49	0.7526	37.45	0.9447	29.01	0.3965	82.70	0.3401
MedGAN [13]	32.54	0.7435	36.96	0.9439	28.95	0.3857	83.72	0.3402
**Ours**	**32.96**	**0.7972**	**33.38**	**0.9529**	**29.22**	**0.4284**	**79.55**	**0.4358**

**Table 3:** Quantitative comparison on the NC→PV synthesis task. Higher PSNR, SSIM, and PCC values indicate better performance, while lower MSE indicates better performance. Bold values indicate the best performance in each column.

Methods	Whole Image				Lesion Area			
	PSNR ↑	SSIM ↑	MSE ↓	PCC ↑	PSNR ↑	SSIM ↑	MSE ↓	PCC ↑
Pix2pix [12]	32.36	0.7430	38.61	0.9467	28.66	0.3754	89.45	0.3701
MedGAN [13]	32.40	0.7319	38.28	0.9462	28.59	0.3552	90.65	0.3600
**Ours**	**32.84**	**0.7870**	**34.45**	**0.9541**	**29.11**	**0.4086**	**80.55**	**0.4979**

**Table 4:** Ablation study on the NC→ART synthesis task. In the “adapted” setting, training freezes the prompts during epochs 1–5 and optimizes them from epoch 6 onward; in the “frozen” setting, the prompts remain fixed throughout training. All prompt-containing variants retain the domain classifier. Higher PSNR, SSIM, and PCC values indicate better performance, while lower MSE indicates better performance. Bold values indicate the best performance in each column.

Methods	Whole Image				Lesion Area			
	PSNR ↑	SSIM ↑	MSE ↓	PCC ↑	PSNR ↑	SSIM ↑	MSE ↓	PCC ↑
No LLTP; no domain classifier	32.46	0.7464	37.63	0.9423	28.75	0.3829	87.46	0.3326
Randomly initialized prompts (adapted)	32.63	0.7585	36.13	0.9364	28.96	0.4182	83.56	0.3970
Qwen3-initialized prompts (frozen)	32.71	0.7531	35.37	0.9316	29.01	0.4063	82.91	0.3569
Phase-swapped Qwen3 prompts (frozen)	32.57	0.7101	36.69	0.8922	28.98	0.3974	83.69	0.2928
**Full: Qwen3-initialized prompts** **(adapted)**	**32.96**	**0.7972**	**33.38**	**0.9529**	**29.22**	**0.4284**	**79.55**	**0.4358**

**Table 5:** Ablation study on the NC→PV synthesis task. In the “adapted” setting, training freezes the prompts during epochs 1–5 and optimizes them from epoch 6 onward; in the “frozen” setting, the prompts remain fixed throughout training. All prompt-containing variants retain the domain classifier. Higher PSNR, SSIM, and PCC values indicate better performance, while lower MSE indicates better performance. Bold values indicate the best performance in each column.

Methods	Whole Image				Lesion Area			
	PSNR ↑	SSIM ↑	MSE ↓	PCC ↑	PSNR ↑	SSIM ↑	MSE ↓	PCC ↑
No LLTP; no domain classifier	32.31	0.7288	38.95	0.9437	28.49	0.3422	92.67	0.3207
Randomly initialized prompts (adapted)	32.53	0.7765	37.00	0.9531	28.68	0.4024	88.91	0.4556
Qwen3-initialized prompts (frozen)	32.65	0.7700	35.88	0.9473	28.93	0.4043	83.89	0.4574
Phase-swapped Qwen3 prompts (frozen)	32.35	0.6791	38.61	0.8897	28.80	0.3745	86.69	0.3086
**Full: Qwen3-initialized prompts** **(adapted)**	**32.84**	**0.7870**	**34.45**	**0.9541**	**29.11**	**0.4086**	**80.55**	**0.4979**

**Table 6:** Downstream slice-level four-class FLL classification performance under three input conditions on the same 127 test slices.

Input Condition	Overall Performance		Class-Wise Recall			
	Accuracy	Macro-F1	Cyst	FNH	HCC	METS
NC only	0.7165	0.6997	0.8824	0.6250	0.7333	0.5417
Synthetic multiphase	0.8504	0.8534	0.8824	0.9167	0.8000	0.8333
Acquired multiphase	0.9134	0.9043	0.9118	1.0000	1.0000	0.6667

Note: The four class-wise columns report recall. NC only denotes [NC,NC,NC]; synthetic multiphase denotes $\left[\mathrm{NC},\hat{\mathrm{ART}},\hat{\mathrm{PV}}\right]$; and acquired multiphase denotes [NC,ART,PV]. Cyst, hepatic cyst; FNH, focal nodular hyperplasia; HCC, hepatocellular carcinoma; METS, liver metastases.

## Data Availability

The data used in this study are not publicly available due to patient privacy and institutional restrictions, but may be available from the corresponding author upon reasonable request and subject to institutional approval.

## Abbreviations

The following abbreviations are used in this manuscript:

ART	Arterial phase
CECT	Contrast-enhanced computed tomography
CT	Computed tomography
GAN	Generative adversarial network
LLM	Large language model
LLTP	LLM-based learnable task prompt
MSE	Mean squared error
NCCT	Non-contrast computed tomography
PCC	Pearson correlation coefficient
PSNR	Peak signal-to-noise ratio
PV	Portal-venous phase
SSIM	Structural similarity index measure

## References

[1] Kim S.W., Kim J.H., Kwak S., Seo M., Ryoo C., Shin C.-I., Jang S., Cho J., Kim Y.-H., Jeon K. (2021). The Feasibility of Deep Learning-Based Synthetic Contrast-Enhanced CT from Nonenhanced CT in Emergency Department Patients with Acute Abdominal Pain. Sci. Rep..

[2] Han S., Kim J.-M., Park J., Kim S.W., Park S., Cho J., Park S.-J., Chung H.-J., Ham S.-M., Park S.J. (2024). Clinical Feasibility of Deep Learning Based Synthetic Contrast Enhanced Abdominal CT in Patients Undergoing Non Enhanced CT Scans. Sci. Rep..

[3] Liu J., Tian Y., Duzgol C., Akin O., Agildere A.M., Haberal K.M., Coskun M. (2022). Virtual Contrast Enhancement for CT Scans of Abdomen and Pelvis. Comput. Med. Imaging Graph..

[4] Pang H., Qi S., Wu Y., Wang M., Li C., Sun Y., Qian W., Tang G., Xu J., Liang Z. (2023). NCCT-CECT Image Synthesizers and Their Application to Pulmonary Vessel Segmentation. Comput. Methods Programs Biomed..

[5] Uhm K.H., Jung S.W., Choi M.H., Hong S.H., Ko S.J. (2022). A Unified Multi-Phase CT Synthesis and Classification Framework for Kidney Cancer Diagnosis With Incomplete Data. IEEE J. Biomed. Health Inform..

[6] Yin J., Peng J., Li X., Ju J., Wang J., Tu H. (2024). Multi-stage Cascade GAN for Synthesis of Contrast Enhancement CT Aorta Images from Non-Contrast CT. Sci. Rep..

[7] Lei Y., Xu L., Wang X., Fan X., Zheng B. (2024). IFGAN: Pre- to Post-Contrast Medical Image Synthesis via Interactive Frequency GAN. Electronics.

[8] Yang Y., Liu J., Chen Q., Han X.H., Li Y., Lin L., Hu H., Chen Y.W. (2024). An improved conditional diffusion model for synthesizing contrast-enhanced computed tomography images. Proceedings of the International KES Conference on Innovation in Medicine and Healthcare.

[9] Ozbey M., Dalmaz O., Dar S.U.H., Bedel H.A., Ozturk S., Gungor A., Cukur T. (2023). Unsupervised Medical Image Translation with Adversarial Diffusion Models. IEEE Trans. Med. Imaging.

[10] Luo Y., Yang Q., Liu Z., Shi Z., Huang W., Zheng G., Cheng J. (2024). Target-Guided Diffusion Models for Unpaired Cross-Modality Medical Image Translation. IEEE J. Biomed. Health Inform..

[11] Kazerouni A., Aghdam E.K., Heidari M., Azad R., Fayyaz M., Hacihaliloglu I., Merhof D. (2023). Diffusion Models in Medical Imaging: A Comprehensive Survey. Med. Image Anal..

[12] Isola P., Zhu J.Y., Zhou T., Efros A.A. (2017). Image-to-Image Translation with Conditional Adversarial Networks. Proceedings of the IEEE Conference on Computer Vision and Pattern Recognition (CVPR).

[13] Armanious K., Jiang C., Fischer M., Küstner T., Nikolaou K., Gatidis S., Yang B. (2018). MedGAN: Medical Image Translation using GANs. arXiv.

[14] Yang Y., Liu J., Zhan G., Chen Q., Wang F., Li Y., Kumar Jain R., Lin L., Hu H., Chen Y.W. (2024). OA-GAN: Organ-aware generative adversarial network for synthesizing contrast-enhanced medical images. Biomed. Phys. Eng. Express.

[15] Emami H., Dong M., Nejad-Davarani S., Glide-Hurst C. (2021). SA-GAN: Structure-Aware GAN for Organ-Preserving Synthetic CT Generation. Proceedings of the Medical Image Computing and Computer-Assisted Intervention (MICCAI).

[16] Chen J., Wei J., Li R. (2021). TarGAN: Target-Aware Generative Adversarial Networks for Multi-modality Medical Image Translation. Proceedings of the Medical Image Computing and Computer-Assisted Intervention (MICCAI).

[17] Liu Z., Lin Y., Cao Y., Hu H., Wei Y., Zhang Z., Lin S., Guo B. (2021). Swin transformer: Hierarchical vision transformer using shifted windows. Proceedings of the IEEE/CVF International Conference on Computer Vision.

[18] Hatamizadeh A., Nath V., Tang Y., Yang D., Roth H.R., Xu D. (2021). Swin UNETR: Swin Transformers for Semantic Segmentation of Brain Tumors in MRI Images. Proceedings of the International MICCAI Brain Lesion Workshop.

[19] Lyu L., Chai S., Liu J., Tomoko T., Qiao X., Chen Y.W. (2023). A 3D Fusion U-Net with Dual CNN and Transformer Encoders for Lung Airway Segmentation. Proceedings of the 2023 IEEE 12th Global Conference on Consumer Electronics (GCCE).

[20] Ding M., Xiao B., Codella N., Luo P., Wang J., Yuan L. (2022). Davit: Dual attention vision transformers. Proceedings of the European Conference on Computer Vision.

[21] Zhou K., Yang J., Loy C.C., Liu Z. (2022). Learning to Prompt for Vision-Language Models. Int. J. Comput. Vis..

[22] Wang Z., Wu Z., Agarwal D., Sun J. (2022). MedCLIP: Contrastive Learning from Unpaired Medical Images and Text. Proceedings of the 2022 Conference on Empirical Methods in Natural Language Processing.

[23] Liu L., Yang X., Lei J., Shen Y., Wang J., Wei P., Chu Z., Qin Z., Ren K. (2024). A survey on medical large language models: Technology, application, trustworthiness, and future directions. arXiv.

